# Senckenberg dogger bank long-term monitoring: First dataset on amphipods

**DOI:** 10.1016/j.dib.2025.111931

**Published:** 2025-07-25

**Authors:** Saeideh Habibi Motlagh, Farzaneh Momtazi, Hanieh Saeedi

**Affiliations:** a Senckenberg Research Institute and Natural History Museum, Senckenberg Data and Modelling Centre and Department of Marine Zoology, Geobiodiversity Informatics, Senckenberganlage 25 60325 Frankfurt am Main, Germany; b Department of Marine Biology, University of Hormozgan, Bandar Abbas, Iran; c Goethe University Frankfurt. Department 15 - Life Sciences, Institute for Ecology, Evolution and Diversity 60438 Frankfurt am Main, Germany

**Keywords:** Long-term monitoring, Biodiversity, Dogger Bank, North Sea, Open-access data, MPA, Amphipoda

## Abstract

This dataset includes unique occurrence records of amphipod specimens collected during the 2024 annual Senckenberg Long-Term Monitoring Project in Dogger Bank (a shallow sand bank in the central North Sea), Cruise DOG24. This cruise was part of an ongoing effort to monitor biodiversity, which has occurred annually from 1991 to 2024 by the Marine Zoology Department at the Senckenberg Research Institute and Natural History Museum. Amphipods, key components of marine benthic ecosystems, were sampled by beam trawl over the Dogger Bank’s stable sandy substrate. A total of 8444 specimens of ten species belonging to 13 families and 14 genera were identified using morphological methods with Leica M60 and DM750 microscopes. This study presents the first species-level identification of benthic amphipods in the Dagger Bank, providing a taxonomically resolved dataset that serves as a reliable identification key for future monitoring efforts in the area. Data were structured and published to the Ocean Biodiversity Information System (OBIS) and the Global Biodiversity Information Facility (GBIF) following the Darwin Core (DwC) standard. This dataset is the first-hand data ever published open-access from the Senckenberg Long Term Monitoring Project since 1991. This dataset also supports a broader research project aimed at (i) revealing the distribution pattern of amphipods in the North Sea, (ii) identifying environmental drivers of species distribution and diversity, and (iii) evaluating the response of the amphipod community to ecosystem changes.

Specifications table

**Table:** 

Subject	Biology
Specific subject area	The specific subject area of this paper includes biodiversity, biogeography, and species identification of Amphipoda
Type of data	Table, Image, Figure, Occurrences, events, and ENV-DATA derived data following the Darwin Core standard
Data collection	The amphipod specimens are catalogued and archived in the Crustacean section, Marine Zoology Department. Unpublished (raw) data were obtained by morphological identification of ethanol-preserved Amphipods. Taxonomic identification was carried out to the lowest achievable level using biological samples collected by beam trawl in the DOG24 Cruise (2024).
Data source location	Institution: Senckenberg Research Institute and Natural History Museum, Department of Marine Zoology, Crustacean Section, Frankfurt am Main, Germany
Data accessibility	Repository name: Ocean Biodiversity Information System (OBIS) and Global Biodiversity Information Facility (GBIF) Data identification number: https://doi.org/10.15468/d7432j [1] Direct URL to data: https://ipt.iobis.org/obis-deepsea/resource?r=amphipoda_doggerbank_monitoring_northsea&v=1.2
Related research article	none

## 1 Value of the Data

• This study presents the first species-level identification of benthic amphipods in the Dogger Bank (a shallow sand bank in the central North Sea), providing a taxonomically resolved dataset that serves as a reliable identification key for future monitoring efforts in the area.
• This species-level identification key allows improved studies of biodiversity assessments and ecological modelling, which are rare in the Dogger Bank benthos datasets.
• Amphipods are sensitive to bottom trawling, hypoxia, and temperature shifts, making this dataset vital for detecting climate and human impact signals.
• A deeper understanding of species distributions and biodiversity of the identified species facilitates effective conservation programs and sustainable marine management in the Dogger Bank, a key Marine Protected Area (MPA).
• This dataset contains all amphipod species recorded in 2024 as part of the Senckenberg Dogger Bank Long-Term Monitoring Program. It is important for comparing changes in the community across monitoring years since 1991.

## 2 Background

Dogger Bank, located in the central North Sea, is one of the largest sandbank areas (300 km) and possesses great ecological significance as a hotspot of marine biodiversity, along with being a nursery area for several species [[2], [3], [4], [5]]. Now, its status as a Marine Protected Area emphasises the importance of long-term monitoring at the species level to assess the effectiveness of implemented conservation actions. However, Centuries of human exploitation have drastically changed the ecological state of the Dogger Bank. This long-term impact is shifting baseline syndrome, which can lead to conservation efforts being based on an inaccurate assessment of the current condition [[6], [7], [8]]. Amphipods play a crucial role in marine benthic food webs and are often the most abundant group of macrozoobenthic taxa in terms of numerical abundance, biomass, and species richness [2]. Amphipods are also sensitive to bottom trawling, hypoxia, and temperature increases, making them useful bioindicators for both human and climate impacts within this delicate ecosystem [2]. This ecological importance, coupled with the Dogger Bank flat and uniform sandy sediment, stable sediment composition, and consistent environmental conditions, is an ideal location for studying benthic community changes over time [[3], [4], [5]].

## 3 Data Description

**Fig. 1:**
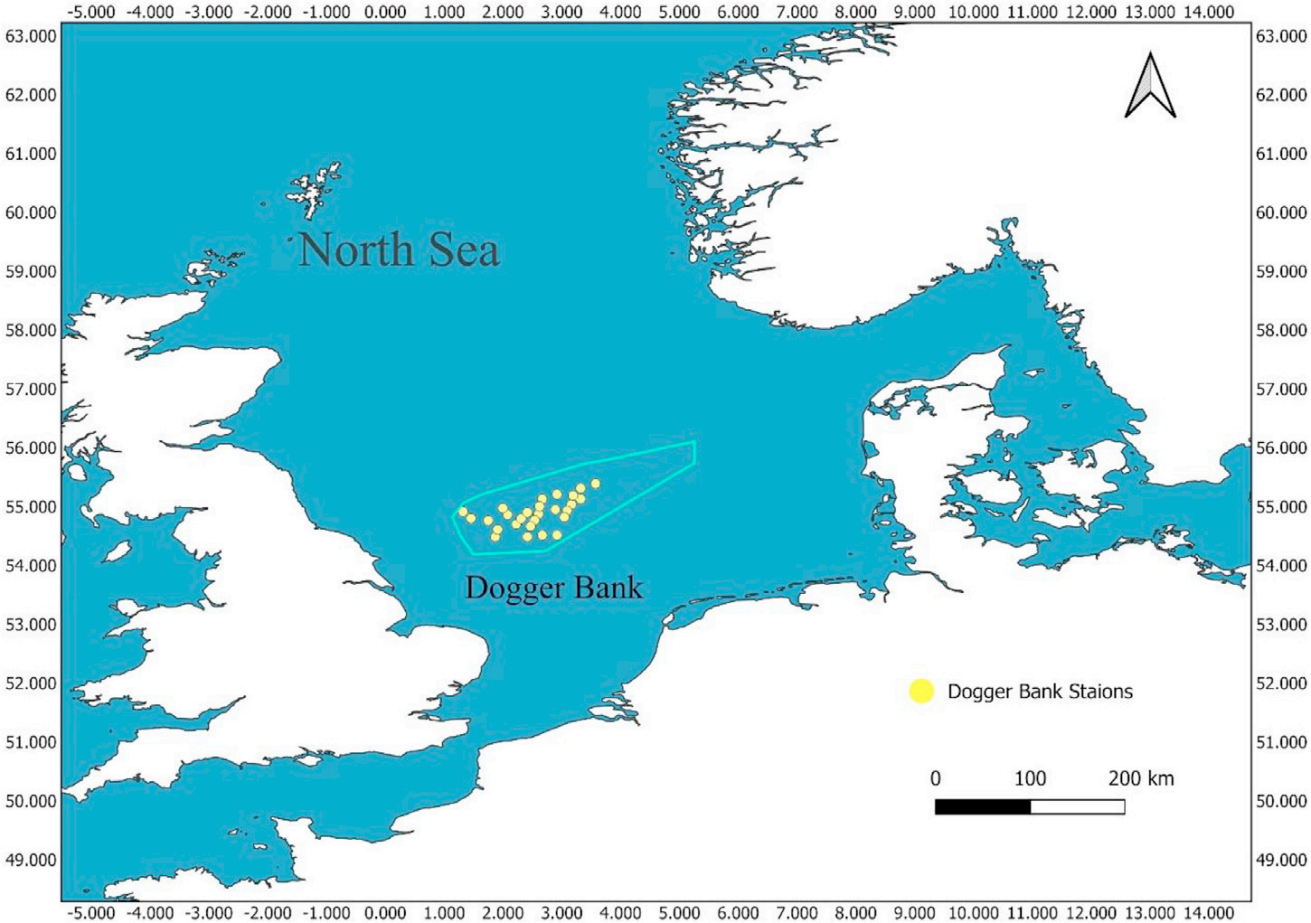
The study region of the cruise DOG24 research expedition, illustrating 27 sampling sites (marked by yellow circles) where amphipod specimens were collected. The light blue polygon shows the Dogger Bank region.

The dataset includes benthic amphipod samples collected in the summer of 2024 at the Dogger Bank region of the North Sea. Sampling took place during the DOG24 scientific research cruise, which is part of the long-term Senckenberg Dogger Bank Monitoring Program. Researchers collected amphipod specimens using a beam trawl (abbreviation Ku) 2 m with 1 cm² mesh size in the cod end, towed for 1 nm at 2 kn of speed, focusing on soft-bottom macrozoobenthos at various predefined sampling stations within the study area [1] (Table 1 and Fig. 1). The abiotic parameters of the study were measured at each station with a CTD probe. The mean temperature was 15.07 °C, the average salinity was 34.26 PSU, and the mean water pressure was 13.65 debar. The sediment composition was predominantly sandy and contained fragment shells.

**Table 1:** This table presents station information, including dates, coordinates, and depths from the DOG 24 cruise research expedition, where amphipods were collected and preserved in ethanol.

Station	Date	Start Coordinates	End Coordinates	Depth [m]	Footprint [WKT]
DOG24:14	2024–07–16	55.10566°N 2.82975° E	54.85105 °N 3.117633° E	25–26	MULTIPOINT* ((54.51063 2.67058), (54.52612 2.68063)
DOG24:30	2024–07–20	55.34416°N 3.53245° E	55.35896° N 3.573217°E	34	MULTIPOINT ((55.20650 2.91947), (55.21538 2.94393)
DOG24:08	2024–07–18	55.51863°N 1.534167 °E	55.49238° N 1.528333° E	30–31	MULTIPOINT ((54.91118 1.32050), (54.89543 1.31700)
DOG24:36	2024–07–21	55.51516° N 3.536417° E	55.50578° N 3.489783° E	29	MULTIPOINT ((55.30910 3.32185), (55.30347 3.29387))
DOG24:29	2024–07–19	55.20953°N 3.114383 °E	55.2265° N 3.1533° E	28–29	MULTIPOINT ((55.12572 2.66863), (55.13590 2.69198))
DOG24:09	2024–07–18	55.6557° N 2.064417° E	55.66103°N 2.111283° E	29–30	MULTIPOINT ((54.99342 1.63865), (54.99662 1.66677))
DOG24:12	2024–07–17	55.16211° N 2.379333 °E	55.1473° N 2.3387° E	23–27	MULTIPOINT ((54.69727 2.22760), (54.68838 2.20322))
DOG24:37	2024–07–21	55.64295° N 3.95695 °E	55.63396° N 3.91075° E	32–33	MULTIPOINT ((55.38577 3.57417), (55.38038 3.54645))
DOG24:21	2024–07–19	55.44741°N 3.0058° E	55.47266° N 3.025167 °E	24–25	MULTIPOINT ((54.86845 2.60348), (54.88360 2.61510))
DOG24:10	2024–07–18	55.54125° N 2.281033 °E	55.52205° N 2.316133° E	26–27	MULTIPOINT ((54.92475 1.76862), (54.91323 1.78968)
DOG24:06	2024–07–17	55.26455° N 2.250417° E	55.24408° N 2.217867° E	27–28	MULTIPOINT (54.75873 1.75025), (54.74645 1.73072)
DOG24:22	2024–07–19	55.27466° N 2.89875 °E	55.30036° N 2.917967 °E	23–24	MULTIPOINT (54.76480 2.53925), (54.78022 2.55078)
DOG24:16	2024–07–20	55.31878° N 2.513333° E	55.3182° N 2.563667° E	23	MULTIPOINT (54.79127 2.30800), (54.79092 2.33820)
DOG24:27	2024–07–20	55.57771° N 3.483217° E	55.59941° N 3.513217 °E	24–25	MULTIPOINT (54.94663 2.88993), (54.95965 2.90793)
DOG24:28	2024–07–19	55.0002° N 3.035917° E	55.02613° N 3.05375° E	29–30	MULTIPOINT (55.00012 2.62155), (55.01568 2.63225)
DOG24:33	2024–07–21	55.0525° N 3.27495° E	55.02516° N 3.2802° E	25–26	MULTIPOINT (55.03150 3.16497), (55.01510 3.16812)
DOG24:17	2024–07–18	55.43116 °N 2.1325° E	55.43366° N 2.1807° E	24–25	MULTIPOINT (54.85870 2.07950), (54.86020 2.10842)
DOG24:18	2024–07–18	55.617° N 2.662617 °E	55.59261° N 2.021717 °E	27	MULTIPOINT (54.97020 1.99757), (54.95557 2.01303)
DOG24:32	2024–07–21	55.59261°N 2.021717° E	55.53641°N 3.11695° E	24–28	MULTIPOINT (54.92703 3.09008), (54.92185 3.11695)
DOG24:35	2024–07–21	55.30816°N 3.322° E	55.28036° N 3.330617° E	30	MULTIPOINT (55.18490 3.19320), (55.16822 3.19837)
DOG24:07	2024–07–18	55.32608° N 1.761867° E	55.31246° N 1.804133° E	25–27	MULTIPOINT ((54.79565 1.45712), (54.78748 1.48248)
DOG24:25	2024–07–20	55.36078 °N 3.073283° E	55.37491° N 3.11675 °E	31–32	MULTIPOINT (54.81647 3.04397), (54.82495 3.07005)
DOG24:20	2024–07–19	55.49695 °N 2.691133° E	55.481 °N 2.730083° E	27	MULTIPOINT (54.89817 2.41468), (54.88860 2.43805)
DOG24:04	2024–07–17	54.8043° N 2.4477° E	54.7892° N 2.40825° E	20–19	MULTIPOINT (54.48258 1.86862), (54.47352 1.84495)
DOG24:03	2024–07–17	55.01175° N 2.51795° E	55.04086° N 2.514633° E	24	"MULTIPOINT ((54.60705 1.91077), (54.62452 1.90878))
DOG24:15	2024–07–19	55.10571°N 2.782783° E	55.10566° N 2.82975°E	20–21	MULTIPOINT (54.66343 2.46967), (54.66340 2.49785)

* Multipoint is a type of geographic geometry that represents multiple distinct points as a single collection of coordinates. https://geojson.org/geojson-spec.html#multipoint.

A total of 8444 amphipod individuals were identified, representing 10 species, 14 genera, and 13 families. Among these, ten species were identified by their full scientific names, while four were identified only to the genus level (sp.). Taxonomic identification was confirmed using the available keys and expert advice of the authors. All records underwent quality checks for consistency and accuracy and were validated.

All occurrence data were formatted according to the Darwin Core standard. This approach ensured interoperability and helped with data integration and mobilisations to OBIS and GBIF, facilitating biodiversity assessments.

This dataset thus provides the first species-level identification and distributions of benthic amphipods in the Dogger Bank. It uses samples from the 2024 Senckenberg Dogger Bank Long-Term Monitoring Program that have not been previously examined. This research establishes a detailed taxonomic reference essential for facilitating on-board species identifications in future annual monitoring activities. This illustrated identification key also addresses the risk of shifting baselines by offering historically informed insights into changes in benthic communities since 1991 (we are examining and identifying the amphipod collections from 1991). This research thus provides essential data for long-term biodiversity monitoring and for evaluating conservation efforts in the Dogger Bank.

## 4 Experimental Design, Materials and Methods

In 2024, researchers collected amphipod specimens using a beam trawl across 27 stations of the Dogger Bank with an average depth of 26.5 m, during cruise DOG24 of the Senckenberg long-term biodiversity monitoring expedition. The Dogger Bank station was plotted in QGIS (Fig. 1).

**Fig. 2:**
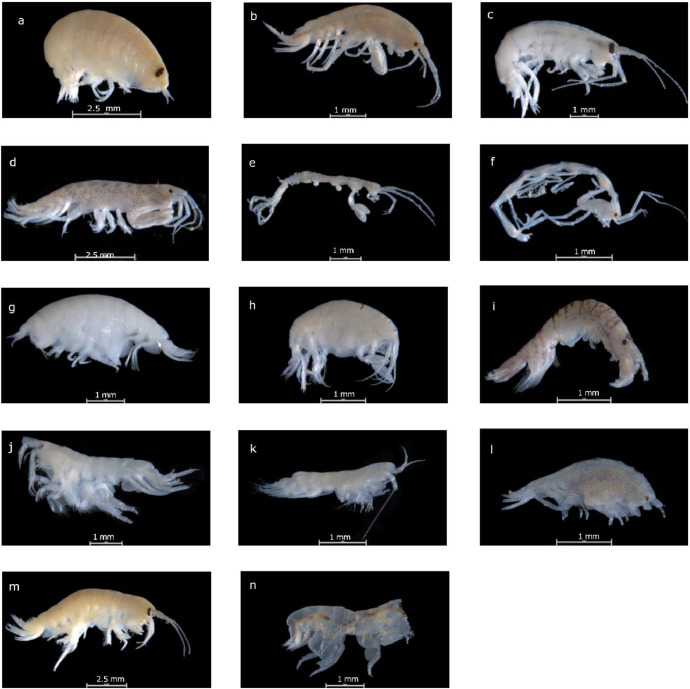
Amphipod specimens from the Dogger Bank (North Sea) region were identified as representative; a) *Scopelocheirus hopei* (A. Costa in Hope, 1851); b) *Abludomelita obtusata* (Montagu, 1813); c) *Nototropis swammerdamei* (H. Milne Edwards, 1830); d) *Aora typica* (Krøyer, 1845); e) *Pariambus typicus* (Krøyer, 1845); f) *Phtisica marina* (Slabber, 1769); g) *Tryphosa nana* (Krøyer, 1846); h (*Stenula solsbergi* (Schneider, 1884); i) *Monocorophium sextonae* (Crawford, 1937); j) *Bathyporeia* sp. (Lindström, 1855); k) *Synchelidium* sp. (G.O. Sars, 1892); l) *Microprotopus maculatus* (Norman, 1867); m) *Gammarus* sp. (Fabricius, 1775); n) *Ampelisca* sp. (Krøyer, 1842).

The samples were preserved in 70 % ethanol. Glycerol was used for dissections and mounting the specimens. Morphological keys were used to identify the species down to the lowest taxonomic level [2,[9], [10], [11], [12]]. The amphipod assemblage was predominantly dominated by species of *Scopelocheirus hopei* (A. Costa in Hope, 1851) (*N* > 1000, 89.61 %), which was by far the most abundant species across nearly all stations. The second most abundant species was *Abludomelita obtusata* (Montagu, 1813) (*N* = 387, 4.58 %) (Fig. 2).

Images were captured using a Lecia stereomicroscope equipped with LAS X software, version 3.5.5.19976. The specimens were deposited in the permanent collection of the Senckenberg Research Institute and Natural History Museum, Marine Zoology Department, Crustacean section, with museum accession numbers ranging from SMF 63,650 to SMF 63,748.

## Limitations

None.

## Ethics Statement

The authors confirm that they have read and comply with the ethical requirements for publication in Data in Brief. We confirm that the present work does not involve human subjects, animal experiments, or data collected from social media platforms.

## CRediT authorship contribution statement

**Saeideh Habibi Motlagh:** Conceptualization, Data curation, Funding acquisition, Investigation, Methodology, Validation, Visualization, Writing – original draft, Writing – review & editing. **Farzaneh Momtazi:** Conceptualization, Investigation, Methodology, Validation, Writing – review & editing. **Hanieh Saeedi:** Conceptualization, Data curation, Funding acquisition, Methodology, Project administration, Resources, Software, Supervision, Validation, Writing – review & editing.

## Data Availability

GBIFSenckenberg Dogger Bank Monitoring Data_Amphipoda_2024 (Original data).
